# NT-proBNP for Heart Failure Screening in Primary Care in an Eastern European Country: What We Know and Proposed Steps

**DOI:** 10.3390/epidemiologia6010002

**Published:** 2025-01-15

**Authors:** Ioana Camelia Teleanu, Anca Mîrșu-Păun, Cristian Gabriel Bejan, Ana-Maria Alexandra Stănescu

**Affiliations:** Department of Family Medicine, “Carol Davila” University of Medicine and Pharmacy, 030167 Bucharest, Romania; ioana.fotino@drd.umfcd.ro (I.C.T.); alexandra.stanescu@umfcd.ro (A.-M.A.S.)

**Keywords:** heart failure, NT-proBNP, primary care

## Abstract

Epidemiological studies indicate that heart failure (HF) prevalence and associated mortality are significantly higher among Eastern European countries as compared to their Western European counterparts. The significant financial burden on the healthcare system matches these sobering data. Thus, efficient programs for patients with HF have been called for. N-terminal prohormone of brain natriuretic peptide (NT-proBNP) represents a widely used, cost-effective, and readily available test that can be used to evaluate HF risk. However, it should not be used as a universal assessment, given the existing variability in proposed cut-off scores for various subgroups of patients. Thus, the clinical context needs to always be considered, and alternative diagnoses need to be ruled out. Based upon evidence from the literature for the above assumptions, the advantages and limitations of using NT-proBNP in primary care settings, along with other HF diagnostic modalities, are discussed in this paper. Also, this paper argues that an effective primary care network, in collaboration with specialist providers, may avoid a delay in HF diagnoses, may help provide on-time treatments, and may ultimately cut unnecessary healthcare expenditures associated with HF hospitalizations. Therefore, the present paper proposes an algorithm for diagnosing HF in primary care settings and discusses specific knowledge and skills that family physicians should be well equipped with in order to successfully respond to the needs of their patients with HF.

## 1. Introduction

Traditionally, heart failure (HF) has been defined as a pathophysiological condition represented by the cardiac pump’s inability to supply sufficient blood flow and to properly oxygenate tissues for their normal activity [1]. Epidemiological data provide evidence that HF represents a worldwide pandemic, given its associated morbidity, mortality, high incidence, and financial burden on national healthcare systems and societies as a whole [2]. Eastern European countries generally record higher prevalence and mortality rates associated with HF than their Western European counterparts. For example, HF prevalence in Romania has been estimated to be 4.7% as compared to the E.U. average of 1.7% [3]. Also, the age-standardized mortality rates associated with cardiovascular diseases are 2.5 times higher in Romania than the E.U. average [3]. In Romania, HF is one of the main causes of hospitalization, and approximately 20% of patients with HF have been readmitted within one year [4]. Possible explanations for these sobering statistics include the following: limited awareness regarding HF, lack of national strategies and guidelines inclusive of quantifiable goals, infrastructure limitations (e.g., patients in rural areas have limited access to specialist healthcare) [3], underutilization of pharmacological treatments, no rigorous follow-up after a hospital discharge, and very low usage of telemedicine practices [5].

The associated costs match the above epidemiological data. A study on data from Romanian patient registries estimated the total yearly expenditures from the national health budget to be EUR 376 million, plus EUR 122 million per year estimated for an average of 13 sick leave days for HF patients of working age, based on the average cost per individual inpatient hospitalization for HF [6]. To this tangible financial burden could also be added the costs associated with a reduced quality of life and emotional impact among patients and their families [7]. Considering this high burden that HF carries on the healthcare system (both financial and human) in addition to the toll on patients’ quality of life, there is an urgent need for coherent national programs for prevention, screening and early detection, diagnosis, and post-discharge monitoring of patients with HF [4].

Given the above-mentioned context, as well as the increasing calls for timely diagnosis and for effective treatment monitoring networks, the present paper proposes an algorithm for HF diagnosis based on current guidelines and updated research regarding the utility of NT-proBNP evaluation. This paper further argues that family physicians may play a significant role in this process, and it outlines the most significant knowledge and skills that family physicians need in order to accomplish this important task.

## 2. Heart Failure (HF): Clinical Aspects

The signs and symptoms most often associated with HF include the following: (a) shortness of breath (manifested as dyspnea, paroxysmal nocturnal dyspnea, exercise intolerance, etc.), (b) tiredness and fatigue, and (c) fluid retention (e.g., ankle swelling, edema, ascites, and weight changes). However, the clinical picture of HF is rather heterogeneous among individual patients, and oftentimes, HF signs and symptoms are considered to be general and unspecific. Moreover, when multiple comorbid conditions are present and/or among elderly patients, differential diagnoses need to be carefully considered and ruled out [8]. Consequently, clinical suspicion of HF might be a provocative endeavor at times rendering added value to objective assessments [9,10].

In the context of HF diagnosis and monitoring, symptom severity has been widely assessed for a long time using the well-known New York Heart Association (NYHA) classification [11]. However, the NYHA class is based on a clinician’s subjective assessment and thus may be subject to inter-rater and/or intra-rater time variability [12].

Signs and symptoms of HF may vary in association with left ventricular ejection fraction (LVEF), which is calculated as stroke volume divided by the end-diastolic volume and reflects the heart’s ability to pump blood [13]. LVEF has been divided into three main categories: (1) reduced EF (HFrEF), defined as LVEF ≤ 40%, (2) preserved EF (HFpEF), defined as LVEF ≥ 50%; and (3) mildly reduced EF (HFmrEF), which includes those patients with LVEF between 41 and 49%. While patients with HFrEF are likely to have experienced a myocardial infarction event or to present ventricular hypertrophy or a bundle branch block [14], those with HFpEF have a close to normal chamber size, a preserved systolic function, and no valvular disease [15]. Determining a patient’s LVEF is essential in terms of diagnosis, treatment, and prognosis. Specifically, while levels of NT-proBNP are typically elevated among patients with HFrEF, they are often in the normal range among patients with HFpEF, thus making the diagnosis of these latter patients more challenging [16]. Also, in accord with current guidelines, patients with HFrEF are more likely to receive more complex drug combinations (of note, the anti-remodeling ARNI and/or SGLT-2 inhibitors have primarily proven their efficacy in the context of HFrEF, and yet their usefulness for all classes of EF is currently discussed).

## 3. Heart Failure (HF) Diagnosis

Early diagnosis is important—and even more so in countries with high HF incidence, given that it allows appropriate treatment interventions and thus helps decrease the risk of progression of HF symptoms and of acute episodes [17]. An HF diagnosis represents a complex, multistep approach that typically requires compounding data on: (1) symptoms typically present among patients with HF, (2) clinical signs typically associated with a diagnosis of HF, and (3) evidence of structural heart disease [18]. Guidelines from the European Society of Cardiology recommend the following: assessment of signs and symptoms, consideration of clinically relevant demographics (such as obesity, hypertension, diabetes, atrial fibrillation, and elderly age), exclusion of alternate diagnoses, and objective assessments—blood tests, ECG, and echocardiography [19]. Utilizing the above HF diagnostic modalities allows healthcare providers to correctly diagnose HF, but also to identify its etiology—which represents a mandatory step for determining appropriate treatments and follow-up procedures. The most frequent cause of HF is coronary artery disease, which may progress to myocardial infarction with a subsequent decreased contractility, activation of neurohormonal compensatory mechanisms, and ultimately hypertrophy and dysfunction. Other common factors associated with HF include poorly managed hypertension (through chronic left ventricular pressure overload that in time affects left ventricular structure and function), valvular pathology, and congenital heart disease (especially in the developing world) [15]. Other (less common causes) include infiltrative cardiomyopathy such as cardiac amyloidosis, peripartum cardiomyopathy, viral myocarditis, and chemotherapy-induced cardiomyopathy [18].

### 3.1. Electrocardiography (ECG)

ECG represents such an objective assessment. It is widely used as an assessment and it represents a quick, highly sensitive—although moderately specific—tool for the diagnosis of HF [8]. More specific ECG patterns linked to heart failure include Q waves and ST elevations (post-acute events), while less specific ECG patterns that may still be linked to HF include left/right bundle brunch blocks, increased QT interval, and also cardiac arrhythmias—(especially ventricular arrhythmias) [20].

### 3.2. Two-Dimensional Doppler Echocardiography

Echo-doppler is typically performed after the initial evaluation of a potential HF. It allows measurements of the left ventricular ejection fraction (LVEF), left ventricular internal dimension and LV mass, septum thickness, LV posterior wall thickness, as well as an evaluation of the valvular function and of the pericardium. Also, echocardiography permits assessing alternative causes of dyspnea such as HF with reduced EF, valve disease, primary pulmonary hypertension, or pericardial effusion [19]. If traditional Doppler echocardiography renders ambiguous findings, other options for evaluating cardiac structure and function are biplane transesophageal echocardiography or radionuclide angiography (MUGA scan). However, these methods are not only costly but also not readily available for some patients (especially those living in rural areas).

### 3.3. NT-proBNP

Although NT-proBNP has sometimes been used as a one-size-fits-all measure, it should be interpreted within a clinical context and based on patients’ demographic and medical profiles [21]. However, its usefulness as an HF diagnostic and monitoring tool has been established, which led to its recognition by most guidelines. Therefore, the 2022 American College of Cardiology/American Heart Association/Heart Failure Society of America (ACC/AHA/NFSA) Joint Committee for Clinical Practice [22], as well as a position paper from the Heart Failure Society of America, Heart Failure Association of the European Society of Cardiology, and Japanese Heart Failure Society [18], clearly outlined the value of using NT-proBNP as an HF diagnosis instrument HF in patients with dyspnea, as well as a monitoring instrument for establishing HF prognosis in hospitalized patients (both are level IA recommendations). NT-proBNP was also recommended for use in patients at risk for developing new-onset HF (recommendation level 2B) [22].

**Figure 1:**
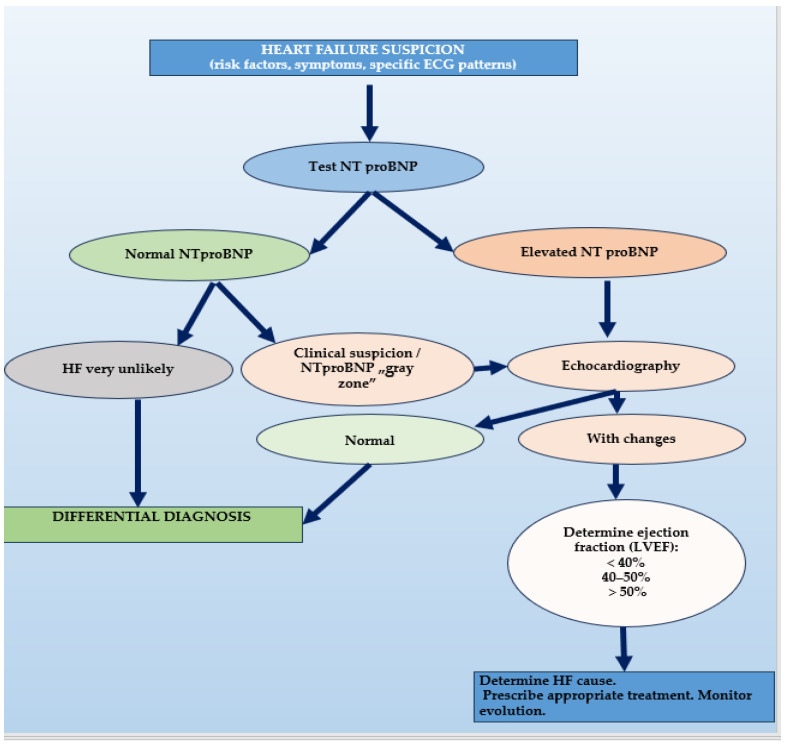
Proposed algorithm for HF diagnosis in primary care, based upon NT-proBNP.

Thus, current guidelines are in agreement that NT-proBNP may be used as part of a reliable, easily available, and cost-efficient HF diagnosis algorithm. Such an algorithm would also incorporate clinical judgment and imaging as required (depicted in Figure 1). Of note, the first steps of this algorithm (i.e., initial clinical assessment and obtaining NT-proBNP levels) may be readily available in primary care to avoid unnecessary and costly specialist referrals.

## 4. The Role of NT-proBNP for HF Diagnosis and Monitoring

Regarding screening and diagnosis of populations at risk for HF, multiple studies have evidenced the significant role that NT-proBNP might have in terms of reduction in HF incidence and HF-associated complications and death. In this context, NT-proBNP has been shown to have a sensitivity of 97% and a specificity of 84% for diagnosing HF in primary care [23]. However, effective and reliable cut-off values are still debated in the literature—particularly when NT-proBNP is used in an outpatient setting [21] and in particular patient populations (for example, NT-proBNP values naturally increase with age and also in association with several comorbidities that are discussed below).

By definition, an optimal cut-off point allows accurately diagnosing most patients (i.e., not missing any patients with HF), which might be the case for a cut-off set too high, while reducing the need for further investigations such as echocardiography for false-positive patients (which might be the case if a cut-off is set too low). Consequently, applied early on in the diagnostic process for patients with suspected cardiac failure, negative NT-proBNP test findings can help rule out a diagnosis of HF and thus avoid unnecessary referral to echocardiography, but if the test result is positive, confirmation by echocardiography will generally be required [24]. First, healthy adults have NT-proBNP levels of less than 70 pg/mL [25]. The European Society of Cardiology (ESC) guideline has set an NT-proBNP cut-off value of 125 ng/mL to rule out HF with slow (i.e., not acute) onset [21] but also recommended NT-proBNP > 220 pg/mL (sinus rhythm) or BNP > 660 pg/mL (in atrial fibrillation) as a major criterion for diagnosing HF [20]. However, this value is not universally accepted. For example, a longitudinal study of primary care patients with signs and symptoms suggestive of HF who were followed over one year examined various cut-off scores for NT-proBNP and determined that an overall score of 150 pg/mL would prevent unnecessary referrals to a cardiology clinic [26]. Another study proposed an absolute NT-proBNP value of 300 pg/mL for ruling out a diagnosis of HF [27], while the National Institute for Health and Care Excellence proposed a general cut-off of 400 pg/mL to rule out HF in untreated individuals [28]. Clearly, while respecting existing guidelines, clinical judgment plays a significant role regarding a diagnosis of HF in primary care settings.

## 5. Variables That Might Alter NT-proBNP Values

Although mostly used for diagnosis purposes, NT-proBNP may be used to monitor ventricular systolic dysfunction as well, with a reported 77% sensitivity and 87% specificity [22]. For example, a longitudinal study on community elderly patients showed that an NT-proBNP value below 190 pg/mL at follow-up was associated with a 50% reduction in HF risk, while an increase of 25% in NT-proBNP values over a 2-year period was associated with a more than double the risk of HF [29]. In addition, NT-proBNP correlates with indicators of left ventricular systolic function, which makes measurements of NT-proBNP useful in adjusting HF treatments [30], although a large meta-analysis found only low-level evidence that treatments informed by serial measurements of NT-proBNP decrease HF mortality [31]. In addition, the most effective follow-up time intervals are still to be determined by future studies [32]. Noteworthily, levels of NT-proBNP might be impacted by different variables.

### 5.1. Age

Since levels of NT-proBNP naturally increase with age, there might be less benefit from using NT-proBNP levels to diagnose or monitor HF among the very old group of patients. Indeed, a meta-analysis study showed that NT-proBNP might not be as useful for patients above 75 years old, compared to patients below 75 years [33]. However, NT-proBNP cut-off scores specific to this age group are still being explored. For example, one study proposed the following age-dependent NT-proBNP cut-off scores to rule out HF: 50 pg/mL for those < 50 years, 75 pg/mL for 50–75 years old, and 250 pg/mL for those over 75 years [34], while a general consensus study proposed NT-proBNP ≥ 125 pg/mL for patients under 50 years, ≥250 pg/mL for patients aged 50–75 years, and ≥500 pg/mL for patients over 75 years [21], while for even older patients (85+ years), an NT-proBNP value of 400 pg/mL was particularly useful as an HF screening instrument for those with an LVEF 40% and below [35]. Of note, these values refer to primary care patients; they are not to be used for acute/emergency department patients.

### 5.2. Renal Function

As renal function declines, levels of NT-proBNP rise because of the reduced clearance by the kidneys; this effect seems to be particularly true for women, more so than for men [36,37]. Therefore, a proposed increase in the NT-proBNP threshold was 35% when eGFR < 30 mL/min/1.73 m^2^ [21]. Based on these findings, the authors established cut-off values for patients with/without renal dysfunction: 100 pg/mL versus 350 pg/mL [38].

### 5.3. Body Mass Index (BMI)

Obesity is associated with lower levels of NT-proBNP, and thus a low threshold should be interpreted with much caution in patients with high BMI [39]. Moreover, NT-proBNP values were predictors of mortality independent of other covariates for all BMI categories except the severely obese individuals, thus raising caution about the predictive value of this marker for this patient population [40]. As an example, cut-offs for effective risk prediction based on NT-proBNP values by BMI and gender groups established in a multicenter study on a very large sample of patients with HF were as follows: for underweight patients with BMI < 18.5 kg/m^2^, NT-proBNP cut-offs were 3385 ng/L for females and 2953 ng/L for men; for normal weight patients (BMI 18.5–24.9 kg/m^2^), NT-proBNP cut-offs were 2728 ng/L for females and 2196 ng/L for men; for patients with class III obesity (BMI ≥ 40 kg/m^2^), NT-proBNP cut-offs were 889 ng/L for females and 631 ng/L for men [40]. Yet, a more general cut-off score of 986 ng/L was proposed by other authors [41].

### 5.4. Gender

Other variables being constant, NT-proBNP values are naturally higher in women than in men. For example, a study conducted in primary care clinics determined that an NT-proBNP cut-off of 100 pg/mL for males and 150 pg/mL for females would be most appropriate [26]. The differences between males and females regarding their NT-proBNP levels are also manifested in different interactions between gender and other variables. For example, older age and smoking seemed to be associated with increased NT-proBNP levels particularly among males, while diabetes seemed to impact NT-proBNP scores (i.e., being associated with elevated NT-proBNP) in females only [42].

### 5.5. Other Factors

*Atrial fibrillation (AF)* is the most common arrhythmia in patients with HF and reduced ejection fraction, and these patients typically present higher levels of NT-proBNP [43]. Patients with AF and preserved ejection fraction (HFpEF) also have higher NT-proBNP levels. For example, levels of NT-proBNP in patients with both paroxysmal or permanent AF were significantly higher than those of patients without AF (in sinus rhythm) [44]. Moreover, among patients with AF, left ventricular ejection fraction and left atrial diameter were independent predictors of NT-proBNP levels [44].

NT-proBNP values might be increased in association with *cardiotoxic drugs* such as oncological medication (anthracyclines, cyclophosphamide, etoposide, and HER-2 antibody trastuzumab) [45,46]. At the same time, the potential effect of more commonly prescribed medications needs to be further evaluated; for example, aspirin and estrogens may be associated with increases in NT-proBNP levels [47]. Taking into account patients’ medications is important, especially when dealing with multiple diagnoses and multiple types of medications.

Other factors potentially associated with increased NT-proBNP levels include the following: *significant pulmonary disease*, *anemia*, *burns*, and *states characterized by high cardiac output* (thyrotoxicosis, sepsis) [48].

## 6. Practical Issues Related to the Use of NT-proBNP for Diagnosing HF—The Role of Family Physicians

The need for increased efforts for HF prevention has recently been emphasized by professional organizations such as the AHA/ACC/HFSA [12]. In Romania also, there is a recognized need to put into action a coordinated national program for cardiovascular disease that would enhance early HF screening and diagnosis—which would further allow a reduction in hospitalization rates and HF-related mortality. Therefore, a statement paper from the Romanian Society of Cardiology (SRC), National Society of Family Physicians (SNMF), and Romanian Heart Foundation (FRI), as well as patients’ associations (C.O.P.A.C.), called for an integrated national program for HF screening, diagnosis, and treatment which could be achieved through coordinated efforts from these entities [4]. Such HF prevention efforts would entail (1) effective identification of at-risk patients as early on as possible and (2) interventions meant to avert further progression of the disease among individuals who have already been diagnosed [2]. Numerous calls have been issued to action for practical steps to be taken to increase its utilization by family/primary care physicians [21].

There are several important reasons why family physicians are expected (as outlined above) to bring a significant contribution to providing healthcare services to patients with HF. First, the role of family physicians is particularly important in those countries and/or regions where access of patients to specialized care is challenging (due to lack of available local healthcare/great physical distance, limited resources to travel, patients’ limited knowledge on steps required to access such medical services, etc.). Patients living in rural areas are particularly confronted with such limitations, especially given that there is a geographical distribution of specialist doctors and dedicated healthcare centers, with most being clustered around large cities and with very scarce resources left for the rural areas. For all such cases, family physicians are expected to play an even more significant role in addressing the healthcare needs of these patients. Second, many Eastern European countries (Romania included) are dealing with a shortage of specialist doctors (the causes are many, including sub-financing of the healthcare system, immigration of specialist doctors, etc.)—a fact that causes the existing specialists to have limited time resources for meeting the needs of great numbers of patients. Thus, they often save their resources for the most challenging cases and hope that the patients without signs and symptoms of severity will be managed through primary care services. Consequently, it is of vital importance that family physicians take the responsibility to diagnose, treat, and monitor these patients, in order to save the time these patients would spend on long waiting lists during which they would be deprived of necessary medication, thus helping patients with HF to avoid episodes of exacerbation. Thirdly, in some Eastern European countries, some cardiologists are still not sufficiently trained to perform echocardiography—until this shortcoming is addressed, primary care medicine may fill the gaps and (in collaboration with specialist physicians) provide an initial diagnosis and treatment based on signs and symptoms and using values of serum NT-proBNP.

Given that family medicine represents a specialty oriented toward primary, secondary, or tertiary prevention [49], family physicians are particularly well equipped to make a significant contribution to the diagnosis and ongoing management of patients with HF [50]. Therefore, the necessary knowledge and skills (summarized in Table 1) include the following:

1. *Have a trained clinical judgment* so that it allows prompt recognition of signs and symptoms associated with HF. This represents a challenging task because of the HF pathophysiological complexity and multitude of risk factors. Also, the initial stages of HF might be pauci-symptomatic (with minor and unspecific signs and symptoms) [2]. For example, signs such as tiredness, ankle swelling, loss of appetite, and even impaired thinking might pass unrecognized, unless specifically reported by the patient. Also, the recognition of clinical symptoms such as elevated jugular venous pressure, tachycardia, hepatomegaly, and oliguria (especially closer to their debut) requires a certain degree of professional experience—especially given that patients usually tolerate these symptoms for long periods of time and only report them to their attending physicians when a decompensation episode is imminent.
2. In addition to recognizing signs and symptoms, family physicians need to be able to *test and exclude differential diagnoses*, which can be challenging, especially among geriatric patients who typically present with multiple comorbidities. For instance, symptoms such as fatigue and dyspnea, or clinical signs such as swollen jugular veins or edema, are not specific to heart failure but may be caused by a range of comorbidities [51].
3. Know how to *properly utilize NT-proBNP,* which has been recognized as a helpful tool for diagnosing and monitoring HF. NT-proBNP increased the diagnostic accuracy of HF by 21% (from 49% to 70%) in a sample of patients who presented with symptoms to their primary care provider [52]. Specific skills include the following: (a) being aware of the diagnostic and monitoring value of NT-proBNP; (b) knowing what NT-proBNP cut-off values are most appropriate for specific groups of patients; (c) understanding that a negative value may rule out HF and yet a positive value does not always mean a diagnosis of HF; (d) being aware of other medical conditions which might be associated with increased levels of NT-proBNP, such as myocarditis, valvular heart diseases, acute coronary syndrome, cardiotoxic drugs, atrial fibrillation, anemia, or sepsis [53]; and (e) being able to correctly interpret NT-proBNP values in a clinical context considering alternate diagnoses. Importantly, however, primary care physicians should keep in mind that currently NT-proBNP testing in primary care is not reimbursed by public insurance companies, and thus, requesting this marker might place a financial burden on some patients with low economic levels.
4. Be able to combine the medical information acquired, in order to *correctly estimate the probability of HF based on a diagnosis algorithm*. In a very large-scale European survey among primary care physicians regarding knowledge and skills for diagnosing HF, physicians in most countries declared unanimously (i.e., approximately 90%) that they would request an ECG for suspected HF; yet, variations were recorded between countries regarding the request for an echocardiography (between 69% in Turkey and 96% in France) [49].
5. Once a diagnosis of HF is made, family physicians should be ready to *initiate HF medication* for those patients without signs and symptoms of severity (i.e., HF class I-II NYHA, normal renal function, no risk factors such as atrial fibrillation, and/or systolic blood pressure over 100 mmHg). Importantly, while some of the standard medications used for HF (i.e., ACEI/ARB, loop diuretics, and beta-blockers) may be prescribed by family physicians as they are compensated, other drugs (especially the SGLT-2 inhibitors and ARNI) must be prescribed by a specialist physician in order to be compensated in Romania. Given that family physicians have continuing access to their patients’ medical histories and are aware of these patients’ comorbidities, they are best suited for evaluating patients’ other medications that might require precaution in HF (for example, corticosteroids may increase fluid and sodium retention).
6. The *management of follow-up care* involves regular meetings with the patient that offer the family physician the opportunity to assess the effectiveness of the treatment, if there are any side effects (for example, if ACEI medication is being well tolerated), and if the dosage needs to be adjusted. On the occasion of these visits, patients may be examined, and signs and symptoms may be explored.
7. Part of a family doctor’s responsibility to follow up on a regular basis with their patients with HF is the task of *treating comorbidities* (which may also represent risk factors), such as hypertension, hyperlipidemia, diabetes, obesity, or smoking. Of note, it is important for the primary care physician to recognize when specialist referrals are needed for more complex cases.
8. Be ready to *discuss lifestyle changes, medication adherence, and symptom monitoring* with patients. Information such as low-sodium diet, fluid restriction, and dietary guidelines including appropriate amounts of protein and lipid intake should be discussed in the primary care setting with patients with HF. Also, self-monitoring tips such as daily weighing, daily BP measurements, and symptom self-monitoring (e.g., ankle swelling, dyspnea, dry cough) should be taught to the patients.
9. *Utilize referral networks* and maintain effective professional communication with specialist physicians in order to allow prompt referral when needed and to coordinate patient-centered and integrated HF care.

In order to illustrate the importance of regular community check-ups for patients with HF, a clinical vignette is presented further. The medical course of this patient clearly illustrates the fact that HF exacerbations and hospital admissions may be prevented by performing regular (ideally monthly) visits to a community health center, in order to assess the need for medication and/or lifestyle adjustments. Noteworthily, NT-proBNP values play a significant role in this process, and the associated costs are significantly lower than those of hospital admission for an HF decompensation episode. Also implied by this case is the importance of primary care physicians also addressing their patients’ medication adherence, as well as dietary and lifestyle recommendations.

 A female patient, 68 years old, priorly diagnosed with HTA, AFib, CHF NYHA class II, presents to the ER complaining of leg edema and dyspnea that first appeared approximately two weeks prior. Her current medication included an ARB, a loop diuretic, NOAC, an antithrombotic agent, and a statin. She exhibited hemodynamic and respiratory stability. NT-proBNP was 8750 pg/mL. She was admitted as an inpatient and remained hospitalized for one week, and was prescribed a higher dose of loop diureticand also a mineralocorticoid receptor antagonist was initiated. NT-proBNP at discharge was 6575 pg/mL. Post-discharge ambulatory monitoring did not occur. The patient returned to the ER after approximately 6 months, with the same presenting concerns. At this second admission, NT-proBNP was 7858 pg/mL. Her treatment dosage was once again adjusted, and she was discharged a second time with NT-proBNP values of 6327 pg/mL This time, however, the patient was monitored on a monthly basis in the community (including her weight, her diet, and her NT-proBNP values), and her medication dosages were adjusted accordingly based on these assessments. She also received advice on lifestyle modifications as required, with the occasion of these monthly check-ups. The patient experienced no more HF decompensation episodes in the past year until the present moment.

## 7. Conclusions

Given the epidemiology of heart failure in our country, there is a clear need for integrated, national HF prevention programs that would include effective diagnosis, treatment, and monitoring strategies. Such efforts focused on prevention through identifying patients at risk for HF, as well as patients with HF at risk for exacerbation, might help preserve healthcare resources and avoid unnecessary human and financial costs. Future studies are needed on large samples of patients regarding effective implementation in primary care of such HF screening programs. Specifically, these studies would examine the best NT-proBNP cut-off values when used in primary care and also would test the effectiveness of the proposed screening model on specific patient groups.

Coordinated efforts are required, involving decision-making forums, local administration, educational entities, family physicians, and last but not least, patients themselves. Decision-making forums may help, for example, by accessing the financial resources necessary to reimburse for NT-proBNP testing of patients with HF. In Romania, NT-proBNP testing is covered by national health insurance only for hospitalized patients but not in primary care. Especially for elderly patients who often need to cover co-payments for several medications, it is sometimes challenging to pay for serum NT-proBNP used for monitoring purposes. However, as previously outlined, it has been shown that such reimbursement would allow primary care physicians to utilize NT-proBNP as an effective tool to monitor patients with HF. This would allow these providers a more effective adjustment of treatments, in order to prevent HF exacerbations—thus avoiding very costly hospitalizations and sparing patients the emotional trouble of going through such an experience.

At the same time, local administration forums may put effort into attracting more doctors (especially in rural areas where oftentimes there is a lack of healthcare workforce) through offering incentives (e.g., a ready-to-use medical facility, certain remuneration advantages, assisting with the procurement of necessary medical equipment, etc.). It is important to mention that currently, in Romania, some of these initiatives are already being discussed in very concrete terms or even in the process of being implemented.

Educational institutions and continuing education forums may ensure that all primary care physicians receive proper training in knowledge and skills required to respond to the needs of their patients with heart failure. Moreover, more continuing education opportunities (such as training sessions, workshops, and professional conferences) may be put in place for physicians who are already practicing, in order to help them sharpen their professional skills. In addition, as previously mentioned, in some countries (Romania included), cardiologists sometimes miss the proper training to perform echocardiography or do not have access to the necessary equipment. Thus, providing ongoing medical education opportunities represents a must.

Practicing (and future) primary care physicians must also be aware of the significant role they may play for patients, such as those with heart failure, and make sure they acquire the knowledge and skills they need to do so in a highly professional manner. The HF diagnosis algorithm for primary care proposed in this paper requires sound clinical judgment—an ability to correctly evaluate signs and symptoms suggestive of HF. This paper provides arguments that family physicians, due to their training, are well fitted for mastering the above clinical skills. Future studies are needed to examine current levels of knowledge and skills that family physicians already possess and to identify any potential areas that need further adjustments through training programs. Also, our proposed model involves creating a network of effective partnerships between primary and specialist care, involving channels for professional communications and an infrastructure put in place to support these collaborations.

Last but not least, patients themselves should be educated and empowered to understand that they also play a significant role in this process, and that only through their willingness to adhere to their medical regiments, understand their own symptoms, implement lifestyle changes, and adhere to HF monitoring plans will they be able to improve their own health condition.

In sum, it is only through coordinated efforts from each one of these entities that a change for the better can become possible, a change materialized as increased effectiveness of healthcare for patients with heart failure and reduced associated costs.

## Figures and Tables

**Table 1:** Knowledge and skills needed for diagnosing HF in primary care, using NT-proBNP.

Domain	Primary Care Physician Professional Abilities	
	*Knowledge*	*Skills*
**Clinical performance**	Recognition of unspecific and general signs and symptoms.	Appropriate examination and follow-up with patients.
**Exclude differential diagnoses**	Sound clinical judgment of patient’s comorbidities.	Request additional examinations when they are indeed necessary.
**Appropriately use NT-proBNP**	Understand what factors might influence NT-proBNP values.	Awareness that a negative value does not automatically rule out an HF diagnosis.
**Use of the diagnosing algorithm to estimate HF probability**	Know the necessary steps to correctly diagnose HF.	Ability to integrate the information and to correctly determine when treatment may be initiated or a specialist referral is needed.
**Initiate HF medication**	Know which medication is appropriate for an individual patient’s diagnosis.	Present to the patient a treatment plan to commonly agree upon.
**Manage follow-up care**	Information regarding possible side effects of the prescribed medication.	Clinical skills necessary to re-evaluate patient’s medical condition.
**Treat comorbidities**	Know what are possible comorbodities (and especially the high-risk ones).	Clinical ability to screen and diagnose these comorbidities.
**Discuss adherence, lifestyle changes, and symptom monitoring**	Knowledge of specific issues to be discussed with patients in this context.	Empathy, sensitivity to patient’s needs, and effective communication skills.
**Maintain a network for professional referral with specialist physicians**	Understand the importance of building and maintaining a network for professional communication.	Appropriate specialist referrals. Attend networking events.

## Data Availability

Not applicable.
